# Poisoning by Ingestion of One Grain of Atropia Sulphate

**Published:** 1888-03

**Authors:** Cassius D. Wescott

**Affiliations:** 567 West Madison Street


### A CASE OF POISONING BY INGESTION OF ONE GRAIN OF ATROPIA SULPHATE.

BY CASSIUS D. WESCOTT, M. D.

[Read before the Chicago Pathological Society.]

The following case occurred during my service as assistant physician in the Illinois Eastern Hospital for the Insane.

At about half past two o'clock on the afternoon of September 21, 1884, Ole A., a terminal dement with melancholy delusions and suicidal tendencies, became possessed of a bottle of eye-lotion containing one grain of sulphate of atropia, and swallowed the contents without the knowledge of his attendant. The carpenter had gone into the attendant's room, leaving the door open. The bottle containing the poison had been carelessly placed upon the top of a wardrobe; the patient had noticed the poison-label upon it, and knowing where it had been placed, slipped into the room when the carpenter's back was turned, took it down, and swallowed the contents, without being observed. I believe he swallowed the entire contents, for the bottle was found empty. The patient told accurately how much he found in it, and said he took it all. His desire to suicide was well known. Furthermore, there is no reasonable doubt that the solution contained the quantity which had been prescribed—one grain.

At 4.30, or two hours afterwards, he was reported as being in a "sort of a fit," and in my temporary absence, was seen by one of my colleagues, to whom he seemed as if emerging from an epileptic seizure. His pupils were widely dilated, his muscles relaxed, and he was in a condition of stupor.

At my evening round at 7 o'clock, when I called for the eye-lotion, and the empty bottle was brought to me, my attention was called to A. He was lying on the floor, apparently unconscious. When shaken vigorously, he aroused and seemed to realize his surroundings, but could say nothing, though making visible effort. His pupils were largely dilated, with mouth and tongue very dry; extremities cold; legs so much paralyzed that he could not stand; face palid; pulse 120, respiration 12, shallow and irregular. So much time had elapsed since the drug was taken that evacuation of the stomach was deemed useless.

I at once administered one-half grain of sulphate of morphia hypodermatically, and had the patient put in bed and surrounded with blankets and hot-water cans, and brisk friction applied to the legs and trunk. The catheter was passed, and nearly three pints of very light-colored clear urine drawn. At 9 o'clock, the pulse had increased in volume and strength, and the surface was warm. The patient made some inarticulate sounds, but his voice was very husky. If not almost constantly shaken and flagellated, he would drop off into a deep sleep, and respiration would become alarmingly slow—10 or 12 to the minute. The following prescription was given:

R. Tr. digitalis, m. xv.
Potassæ nit., gr. x.
Spirits frumenti, ℥ ii.
M. S. At once.

At 9.30, paralysis had almost entirely disappeared, and he was walked between two attendants until 11 p. m., but the tendency to sleep was so great that occasional flagellations were necessary to keep him awake, even while walking. The pulse was 150; temperature 101° F.; respiration 20 and regular. The tongue had become moist, and the patient would reply to questions in monosyllables, and swear at the attendants when they slapped him unusually hard. He complained of some headache. Digitalis, nitre, and whisky were repeated, and the catheter passed, but only a small quantity of dark-colored urine was obtained. The patient was then put in bed, and kept awake by occasional flagellations until 1 a. m., when he was intrusted to the care of two attendants, with instructions to keep him moving and give him two drachms of whisky every hour. They reported having walked him until 4 a. m., when they allowed him to rest, and with an occasional shake kept him awake. At the rising hour, 5 a. m., he dressed himself and went to breakfast with the others of his ward. At 6 o'clock, his pupils were largely dilated, but he looked and acted as well as usual, said he had no headache and felt well. As the urine had not been passed voluntarily, the catheter was resorted to, and twelve ounces of very dark-colored urine were drawn. The pupils remained dilated for some days, but otherwise nothing unusual was observed in the patient physically; mentally, he seemed brighter and more cheerful for some weeks.

There was no delirium observed at any time, and only occasional muscular twitchings during the early part of the night. The fit reported by the attendants before the patient was seen by a physician was described as "like an epileptic fit."

The case seems of interest because of the large amount of atropia taken, and the rapidity and completeness of the recovery. Dr. H. P. Loomis, of New York City, reports a case (*Medical Record*, February 28, 1885), in which a similar quantity was taken, with symptoms fully as alarming and more serious after effects, but ultimate recovery. He says he could find only one case (in either English or American journals) in which so large a quantity of the salt was taken and recovery occurred. This case was reported in the *Medical Times and Gazette*, July 6, 1865. I have searched the literature at my command with similar results, and hence feel justified in offering this case for your discussion.

567 West Madison Street.

